# Elucidation of Molecular Mechanisms of Physiological Variations between Bovine Subcutaneous and Visceral Fat Depots under Different Nutritional Regimes

**DOI:** 10.1371/journal.pone.0083211

**Published:** 2013-12-09

**Authors:** Josue Moura Romao, Weiwu Jin, Maolong He, Tim McAllister, Le Luo Guan

**Affiliations:** 1 Department of Agricultural, Food and Nutritional Science, University of Alberta, Edmonton, Alberta, Canada; 2 Lethbridge Research Centre, Agriculture and Agri-Food Canada, Lethbridge, Alberta, Canada; Moffitt Cancer Center, United States of America

## Abstract

Adipose tissue plays a critical role in energy homeostasis and metabolism. There is sparse understanding of the molecular regulation at the protein level of bovine adipose tissues, especially within different fat depots under different nutritional regimes. The objective of this study was to analyze the differences in protein expression between bovine subcutaneous and visceral fat depots in steers fed different diets and to identify the potential regulatory molecular mechanisms of protein expression. Subcutaneous and visceral fat tissues were collected from 16 British-continental steers (15.5 month old) fed a high-fat diet (7.1% fat, n=8) or a control diet (2.7% fat, n=8). Protein expression was profiled using label free quantification LC-MS/MS and expression of selected transcripts was evaluated using qRT-PCR. A total of 682 proteins were characterized and quantified with fat depot having more impact on protein expression, altering the level of 51.0% of the detected proteins, whereas diet affected only 5.3%. Functional analysis revealed that energy production and lipid metabolism were among the main functions associated with differentially expressed proteins between fat depots, with visceral fat being more metabolically active than subcutaneous fat as proteins associated with lipid and energy metabolism were upregulated. The expression of several proteins was significantly correlated to subcutaneous fat thickness and adipocyte size, indicating their potential as adiposity markers. A poor correlation (r=0.245) was observed between mRNA and protein levels for 9 genes, indicating that many proteins may be subjected to post-transcriptional regulation. A total of 8 miRNAs were predicted to regulate more than 20% of lipid metabolism proteins differentially expressed between fat depots, suggesting that miRNAs play a role in adipose tissue regulation. Our results show that proteomic changes support the distinct metabolic and physiological characteristics observed between subcutaneous and visceral adipose tissue depots in cattle.

## Introduction

In the past decade, adipose tissue has received increasing attention since fat not only aids in the regulation of energy balance, but also plays an important role in endocrine function [1,2]. Adipose tissue dysfunction has also become an important health concern and obesity is now considered as an epidemic condition [3] with more than 1.5 billion people worldwide being overweight or obese [4]. One of the concerns in the development of obesity is the increase of the consumption of high fat foods; therefore efforts have been made to increase the leanness of beef [5,6]. Meat is an important protein source and the world meat demand is projected to increase by 73% from 2010 to 2050 [7]. Since fat is an important component in animal productivity and meat quality [8,9], it is necessary to improve our understanding on regulation of adipogenesis in beef in order to provide meat with lipid profiles that are desirable for human consumption. 

Adipogenesis is an essential biological process in mammals, which involves the development of mature adipocytes from preadipocytes [10]. This process modulates the adiposity of individuals and can be influenced by various factors such as diet, fat depot, age and genetics [11-13]. Adipogenesis is regulated by transcription factors such as peroxisome proliferator-activated receptor gamma (PPARγ), members of CCAAT/enhancer binding proteins, kruppel-like factors, and sterol regulatory element-binding proteins (SREBP), which control the expression of adipogenic genes that participate in the differentiation of adipocytes [14,15]. Up to date, our understanding on molecular regulation of adipogenesis is based mainly on information obtained from gene expression studies at the mRNA level [16]. However, transcript levels do not necessarily correlate well with protein expression [17-19] and therefore phenotype. Recently, it has become evident that not only transcription factors but also microRNA (miRNAs) [20-22] and epigenetic mechanisms are implicated in the regulation of adipose tissue metabolism [23]. Post-transcriptional regulatory mechanisms such as RNA interference as carried out by miRNAs or small RNAs may repress the translation of mRNAs into proteins [24]. As the correlation between mRNA levels and protein expression is moderate [17-19], characterization of translated proteins may be a better predictor of phenotype.

To date, few studies have attempted to study the regulation of bovine adipogenesis at the protein level. Using 2D electrophoresis and MS analysis, 13 differentially expressed proteins were identified between steers with high or low subcutaneous fat thickness [25]. Differentially expressed proteins were also identified between preadipocytes and mature adipocytes, and also among adipocytes from different fat depots (omental, subcutaneous and intramuscular) [26]. However, the influence of diet and fat depots on proteome of bovine adipose tissue *in vivo* has not been investigated. Therefore, the aim of our study was to characterize the proteomic profile of subcutaneous and visceral adipose tissues of beef steers fed a high or a low fat level diet and to identify the potential regulatory molecular mechanisms of protein expression.

## Results

### Location and types of proteins expressed in bovine adipose tissue

**Figure 1:**
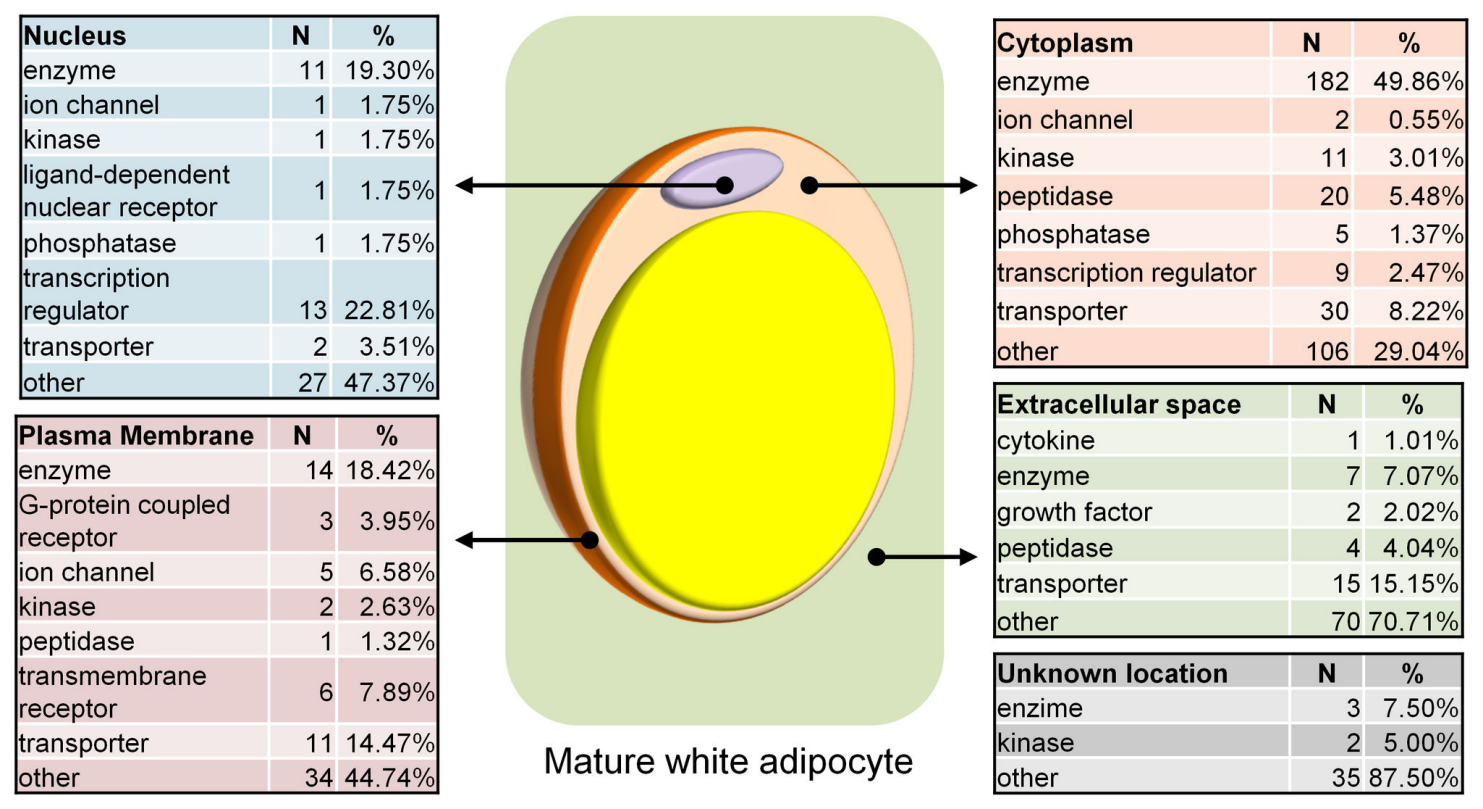
Types and cellular location of proteins identified from bovine adipose tissue. Mature adipocytes represent the main cellular type in adipose tissue, but other cell types including macrophages, endothelial cells, preadipocytes and stem cells may also be present in adipose tissue.

A total of 682 proteins were identified and quantified with at least one unique peptide in all experimental groups: Control (2.7% fat) diet/Subcutaneous adipose tissue (C.SAT), Control diet/Visceral adipose tissue (C.Vat), High fat (7.1% fat) diet/Subcutaneous adipose tissue (HF.SAT) and High fat diet/Visceral adipose tissue (HF.VAT). Among these proteins, 637 were classified into a main protein category based on their function and into a cellular location including the nucleus (n=57), cytoplasm (n=365), plasmatic membrane (n=76), extracellular space (n=99) or unknown location (n=40) according to the Ingenuity Knowledge Base using Ingenuity Pathway Analysis Software Package (IPA^®^) (Figure 1). The function and/or location of the remaining proteins in our study could not be annotated, and therefore only these 637 proteins were subjected downstream analysis. 

### Functional proteome of bovine adipose tissue

**Figure 2:**
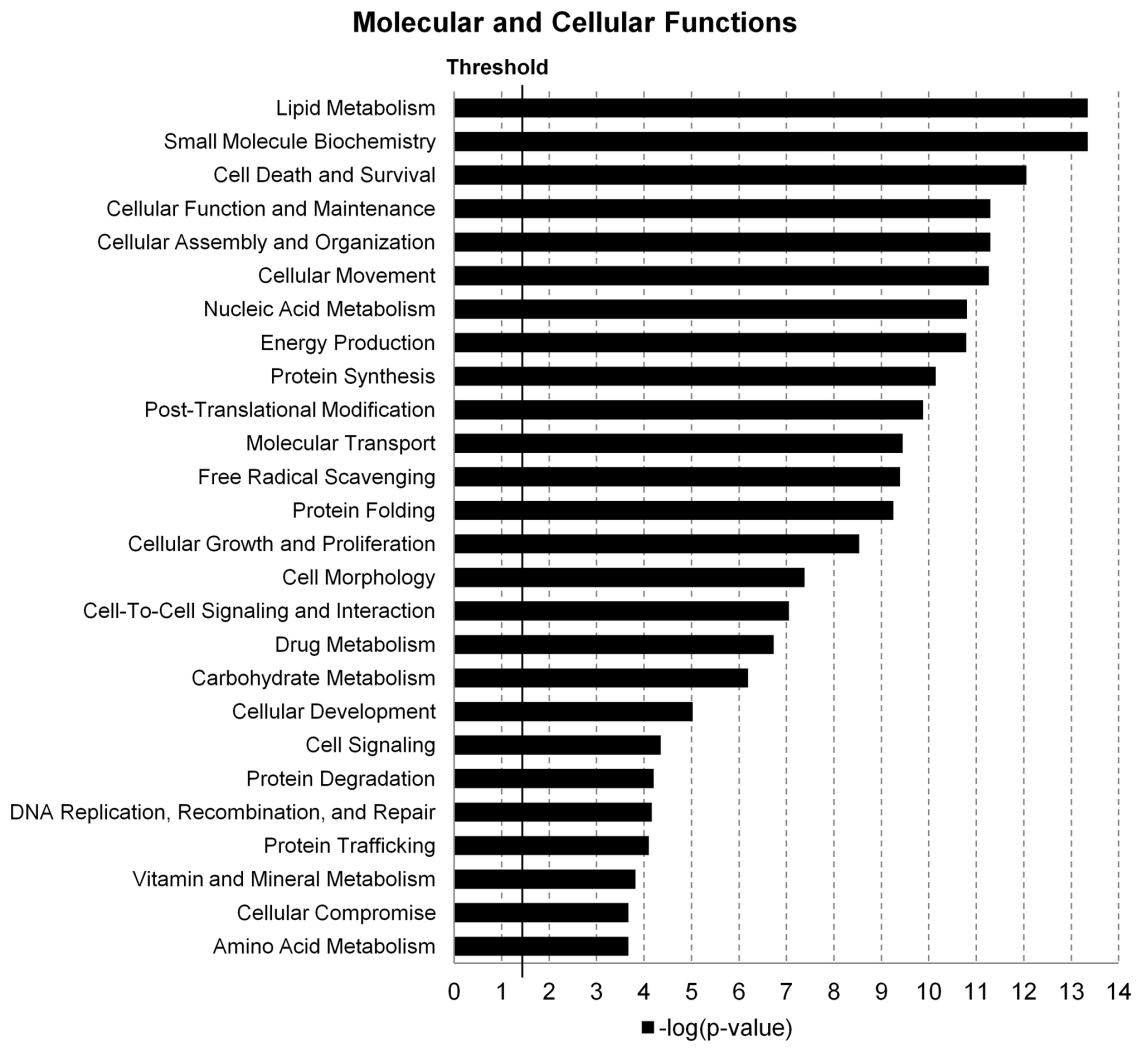
Molecular and cellular functions in bovine adipose tissue. The likelihood of the association between the proteins in the dataset and a biological function is represented as –log(p-value), with larger bars being more significant than shorter bars. The vertical line indicates the cutoff for significance (p-value of 0.05).

The functional analysis identified the most relevant biological functions of the identified proteins dataset. Proteins were associated with biological functions according to the Ingenuity Knowledge Base (IPA^®^) and a Right-tailed Fisher’s exact test calculated a p-value determining the probability that each function assigned to that dataset is due to chance alone. Twenty six biological functions of proteins at molecular and cellular levels were (p<0.05 or –log(p-value)>1.3) identified in bovine adipose tissue. Lipid metabolism, small molecule biochemistry, cell death and survival, cellular function and maintenance, and cellular assembly and organization were the five most relevant predicted functions (Figure 2). 

### Protein expression changes to adipose depot and diet

**Figure 3:**
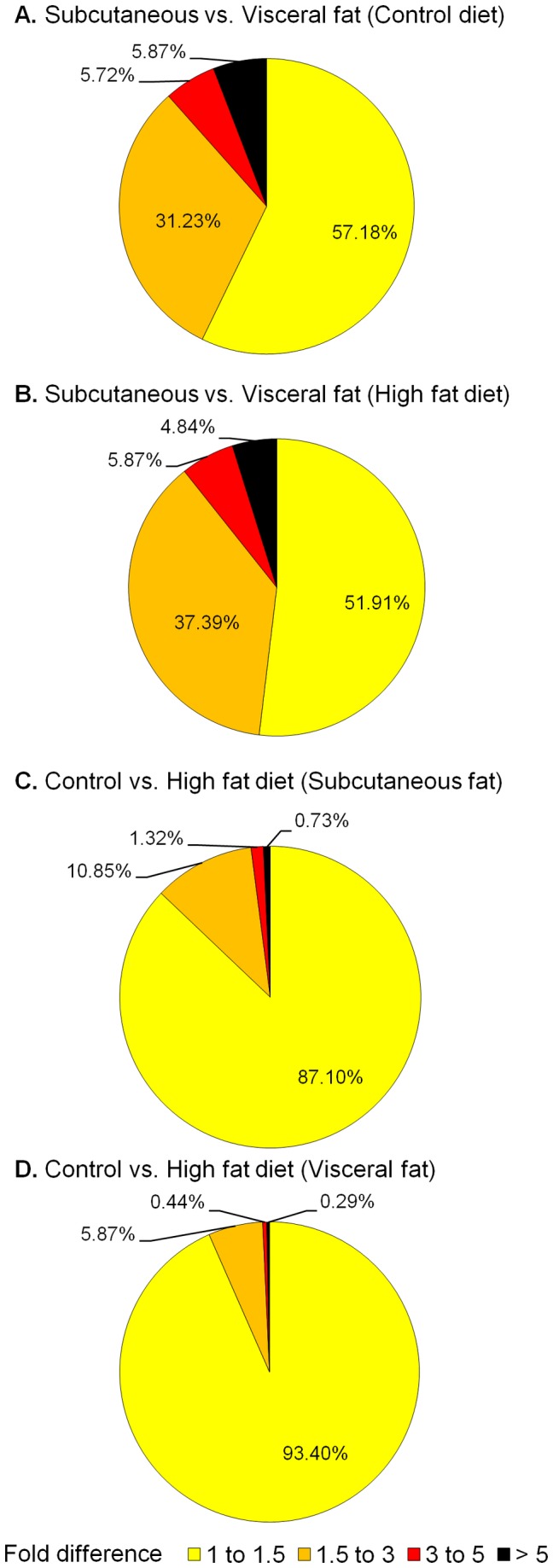
Proteome expression changes due to fat depot and diet. Changes in expression of 682 proteins are represented by fold change differences (1 to 1.5, 1.5 to 3, 3 to 5, and >5) in adipose tissue collected from different depots in steers fed control of high fat diets: A (Subcutaneous vs. visceral adipose tissue in steers fed Control diet), B (Subcutaneous vs. visceral adipose tissue in steers fed high fat diet), C (Control vs. high fat fed steers using subcutaneous fat tissue), and D (Control vs. high fat fed steers using visceral adipose tissue).

Fat depot and diet influenced protein expression; however a two-factor ANOVA analysis revealed that there was minimal interaction between diet and fat depot on protein expression. The expression of 51.0% of proteins was affected by fat depot and 46.9% was not affected by either diet or depot (Table 1). Between subcutaneous and visceral adipose tissues, 57.2% of proteins exhibited less than a 1.5 fold difference (FD) (-1.5 < FD < +1.5) in expression, while 42.8% varied more than 1.5 FC (-1.5 ≤ FD ≥ +1.5) in steers fed the control diet (Figure 3A). In steers fed the high fat diet, 51.9% of proteins varied less than 1.5 FD (-1.5 < FD < +1.5) in expression while 48.1% exhibited expression above this level (-1.5 ≤ FD ≥ +1.5) (Figure 3B). When the control and high fat diet were compared, most proteins (87.1%) changed less than 1.5 fold change (FC) (-1.5 < FC < +1.5) in subcutaneous adipose tissue (Figure 3C), while 93.4% of proteins exhibited less than 1.5 FC (-1.5 < FC < +1.5) in visceral fat tissue (Figure 3D). Expression values for all proteins according to groups are shown in Table S1. 

**Table 1:** Effects of fat depot, diet and fat depot x diet interaction on protein expression.

**% Proteins**	**# Proteins**	**Fat depot**	**Diet**	**Interaction FxD**
47.80%	326	<0.01	NS	NS
46.92%	320	NS	NS	NS
3.08%	21	<0.01	<0.01	NS
1.91%	13	NS	<0.01	NS
0.15%	1	<0.01	<0.01	<0.01
0.15%	1	NS	<0.01	<0.01

Fat depot, diet and their interaction have a significant effect on protein expression when a p-value <0.01 is assigned, while NS indicates no significant effect (p>0.01). P-values were calculated for Two-Way ANOVA analysis.

### Functional analysis of proteins differentially expressed by fat depot or diet

Functional analysis of differentially expressed (DE) proteins (-1.5 ≤ FC ≥ +1.5 and p-value<0.05) was undertaken according to the fat depot and diet fed. In total 240 DE proteins were identified between C.SAT and C.Vat, 243 between HF.SAT and HF.VAT, 32 between C.SAT and HF.SAT, and 17 between C.Vat and HF.VAT (Table 2). The top 5 molecular and cellular functions of DE protein sets from the above comparisons are listed in Table 2. Comparison of DE proteins between subcutaneous and visceral adipose tissues, irrespective of the diet, showed lipid metabolism as one of the most relevant functions. The Downstream Effects Analysis (IPA^®^) revealed that the visceral adipose tissue of steers fed control diet exhibited an increase in the activity of oxidation of lipids, oxidation of fatty acids and synthesis of lipids as compared to subcutaneous adipose tissue (z-score > 2). Similarly, steers fed the high-fat diet exhibited increased activity of lipid synthesis and the efflux of cholesterol in visceral fat than in subcutaneous fat. Furthermore, the number of upregulated proteins involved in lipid metabolism in subcutaneous fat was less than half of that in visceral fat with both diets. A total of 36 proteins were affected by diet (p<0.01). Among these, 6 proteins are known to participate in lipid metabolism and expression of three was notably higher in steers fed high fat diets: stearoyl-CoA desaturase (delta-9-desaturase) (SCD), apolipoprotein C-III (APOC3), and annexin A6 (ANXA6). While perilipin 1 (PLIN1), malate dehydrogenase 1, NAD (soluble) (MDH1), and integrin, alpha 6 (ITGA6) were expressed at higher levels in steers fed the control diet (Table S1). Due to limited number of DE proteins between control and high-fat diets, the downstream effects analysis was not able to predict activation states (increased or decreased) for biological functions influenced by diet in these datasets.

**Table 2:** Top 5 categories of molecular and cellular functions of differentially expressed proteins according to adipose tissue depot and diet.

Comparison	Functional Category	Functions involved	p-value range	DE Proteins	Up-regulated	Down-regulated
C.SAT / C.Vat (n=252)	Energy Production	11	1.48x10^-9^ - 2.21x10^-3^	34	7	27
	Lipid Metabolism	43	1.48x10^-9^ - 4.23x10^-3^	51	16	35
	Small Molecule Biochemistry	62	1.48x10^-9^ - 4.78x10^-3^	72	19	53
	Post-Translational Modification	7	5.17x10^-9^ - 4.06x10^-3^	19	5	14
	Cellular Assembly and Organization	22	2.12x10^-8^ - 6.22x10^-3^	55	30	25
HF.SAT / HF.VAT (n=270)	Cell Morphology	14	3.20x10^-12^ - 5.64x10^-3^	42	18	24
	Cellular Assembly and Organization	22	3.20x10^-12^ - 5.88x10^-3^	65	34	31
	Protein Synthesis	7	1.12x10^-10^ - 4.62x10^-3^	38	9	29
	Cellular Function and Maintenance	17	2.02x10^-9^ - 5.43x10^-3^	87	41	46
	Lipid Metabolism	48	6.97x10^-9^ - 6.36x10^-3^	60	16	44
C.SAT / HF.SAT (n=39)	Cell Morphology	10	3.03x10^-4^ - 3.76x10^-2^	5	1	4
	Lipid Metabolism	29	9.28x10^-4^ - 4.59x10^-2^	5	1	4
	Small Molecule Biochemistry	40	9.28x10^-4^ - 4.59x10^-2^	12	2	10
	Carbohydrate Metabolism	7	1.74x10^-3^ - 1.04x10^-2^	3	2	1
	Cell-To-Cell Signaling and Interaction	24	1.74x10^-3^ - 4.76x10^-2^	12	3	9
C.Vat / HF.VAT (n=15 )	Cellular Development	26	4.41x10^-5^ - 4.70x10^-2^	8	3	5
	Cellular Growth and Proliferation	19	4.41x10^-5^ - 2.36x10^-2^	9	3	6
	Cellular Movement	27	2.49x10^-4^ - 4.29x10^-2^	6	2	4
	Cellular Assembly and Organization	44	4.84x10^-4^ - 4.70x10^-2^	8	3	5
	Cellular Function and Maintenance	25	4.84x10^-4^ - 4.70x10^-2^	8	2	6

C.SAT: Control diet/ Subcutaneous adipose tissue, C.Vat: Control diet/Visceral adipose tissue, HF.SAT: High fat diet/Subcutaneous adipose tissue, and HF.VAT: High fat diet/Visceral adipose tissue. Functions involved: number of detected molecular and cellular functions that are involved with the main functional category. P-value range: presents the range of p-values from lowest to highest of functions for each functional category, being p<0.05 significant. DE proteins: amount of differentially expressed proteins between group comparisons that participate in respective function.

### Relationship between protein expression and fat traits

Subcutaneous fat thickness and adipocyte size are two measures of adiposity with thickness ranging from 11 to 27 mm, and adipocyte size ranging from 118 to 163µm among steers in the current study. Pearson correlation between these adiposity traits and expression of proteins identified the top 10 significant (p<0.05) correlations (Tables 3 and 4). Three out of the top ten proteins correlated with subcutaneous fat thickness are involved in lipid metabolism (DBI, FABP5 and NQO1), while only one of the top ten proteins correlated to adipocyte size is involved with lipid metabolism (FABP4). Subcutaneous fat thickness and adipocyte size were positively correlated (r=0.377), however, all the top 10 proteins that correlated with each of these respective traits were different.

**Table 3:** Top 10 positive correlations between protein expression and thickness of subcutaneous fat.

**Protein ID**	**Gene ID**	**Description**	**R**	**p-value**
Q3SYV4	CAP1	adenylate cyclase-associated protein 1	0.628	0.009
F1MHB8	QPRT	quinolinate phosphoribosyltransferase	0.626	0.009
P07107	DBI	diazepam binding inhibitor	0.611	0.012
Q3ZBH2	NQO1	NAD(P)H dehydrogenase, quinone 1	0.578	0.019
P55052	FABP5	fatty acid binding protein 5	0.577	0.019
E1BEL7	HSPB1	heat shock 27kDa protein 1	0.575	0.020
F1MNT4	LAMB1	laminin, beta 1	0.574	0.020
A8E641	DPYSL5	dihydropyrimidinase-like 5	0.557	0.025
Q3ZBD7	GPI	glucose-6-phosphate isomerase	0.554	0.026
Q32KL2	PSMB5	proteasome (prosome, macropain) subunit, beta type, 5	0.553	0.026

Correlation is significant at p<0.05

**Table 4:** Top 10 positive correlations between protein expression and adipocyte size.

**Proteins ID**	**Gene ID**	**Description**	**R**	**p-value**
F1MBU7	CDCA7L	cell division cycle associated 7-like	0.673	0.004
E1B8H0	PEAK1	NKF3 kinase family member	0.594	0.015
A6QR11	NELL2	NEL-like 2 (chicken)	0.581	0.018
P11181	DBT	dihydrolipoamide branched chain transacylase E2	0.559	0.025
P00129	UQCRB	ubiquinol-cytochrome c reductase binding protein	0.544	0.029
F1MHQ4	FABP4	fatty acid binding protein 4, adipocyte	0.541	0.031
G5E5C8	TALDO1	transaldolase 1	0.526	0.037
E1BN43	ANKRD28	ankyrin repeat domain 28	0.522	0.038
F1MDH3	TLN1	talin 1	0.513	0.042
Q17QZ6	SDPR	serum deprivation response	0.510	0.044

Correlation is significant at p<0.05

### Transcriptional regulation of adipose tissue

#### The Upstream Regulator Analysis (IPA^®^) revealed that 131 transcription factors

(TFs) may regulate the transcription of genes that resulted in the translation of proteins that were differentially expressed between subcutaneous and visceral adipose tissue depots (n=252) in steers fed the control diet. Similarly, 145 TFs may be associated with differential regulation of proteins expressed in adipose tissues (n=270) depots from steers fed the high-fat diet. Nine TFs relevant (p<0.05) for each DE protein dataset had their activation state (increased or decreased) significantly predicted (z-score >2 or <-2) based on the expression of the DE proteins they regulate (Table 5). Most of these TFs (8 out of 9) were in common for steers fed both diets and had the same predicted activation state. Only 1 out of 9 TFs was predicted as being increased in subcutaneous fat whereas 8 were predicted to exhibit increased expression in visceral adipose tissue.

**Table 5:** Predicted transcription factors regulating DE proteins between adipose tissue depots.

		C.SAT x C.Vat				HF.SAT x HF.VAT			
Transcription factors	Description	State C.Vat	z-score	Overlap p-value	Target proteins	State HF.VAT	z-score	Overlap p-value	Target proteins
GATA4	GATA binding protein 4	↓	2.236	4.85x10^-4^	6	↓	2.433	1.39x10^-4^	7
KLF15	Kruppel-like factor 15	-	-	-	-	↑	2.017	1.12x10^-5^	6
MEF2C	myocyte enhancer factor 2C	↑	2.433	8.40x10^-5^	6	↑	2.433	1.70x10^-4^	6
NFKB1	NFKB1 nuclear factor of kappa light polypeptide gene enhancer in B-cells 1	↑	2.000	3.49x10^-2^	6	↑	2.000	6.57x10^-2^	8
NRIP1	nuclear receptor interacting protein 1	↓	-2.236	5.34x10^-4^	5	↓	-2.646	8.32x10^-7^	8
PPARA	peroxisome proliferator-activated receptor alpha	↑	2.049	1.92x10^-15^	29	↑	2.692	8.33x10^-15^	30
PPARG	peroxisome proliferator-activated receptor gamma	↑	3.082	6.18x10^-16^	29	↑	3.397	1.89x10^-14^	29
PPARGC1A	peroxisome proliferator-activated receptor gamma, coactivator 1 alpha	↑	2.739	1.47x10^-11^	16	↑	3.297	5.77x10^-15^	20
SPDEF	SAM pointed domain containing ets transcription factor	↑	2.236	1.10x10^-3^	5	-	-	-	-
TBX5	T-box 5	↑	2.236	6.49x10^-5^	5	↑	2.236	1.19x10^-4^	5

C.SAT= Control diet/Subcutaneous adipose tissue, C.Vat= Control diet/Visceral adipose tissue, HF.SAT= High fat diet/Subcutaneous adipose tissue, and HF.VAT= High fat diet/Visceral adipose tissue. State ↑ indicates increased activation of the transcription factor in C.VAT or HF.VAT and ↓ indicates decreased activation status. Z-score: indicates the level of confidence to which a transcription factor has its activation status predicted as increased or decreased, being z-score >2 or <-2 significant. Overlap p‐value: analyze whether there is a statistically significant overlap between the dataset proteins and the genes that are regulated by a transcription factor with p<0.05 being significant. Target proteins: indicates the amount of proteins in the experiment dataset that may be regulated by the transcription factor.

A total of 23 TFs were predicted (p<0.05) to regulate 39 proteins differentially expressed between diets in subcutaneous fat, while 63 TFs were predicted (p<0.05) to control 15 proteins differentially expressed between diets in visceral fat. However, due to the limited number of DE proteins by diet none of these transcription factors obtained predictions for their activation state.

### Translation: messenger RNA to protein expression

Nine genes Acyl-CoA oxidase 2, branched chain (ACOX2), aminolevulinate dehydratase (ALAD), ATP synthase, H+ transporting, mitochondrial F1 complex, gamma polypeptide 1 (ATP5C1), ELOVL fatty acid elongase 6 (ELOVL6), fatty acid binding protein 4, adipocyte (FABP4), fatty acid synthase (FASN), fibrillin 1 (FBN1), glycerol-3-phosphate dehydrogenase 1 (soluble) (GPD1), thyroid hormone responsive (THRSP) were selected to evaluate their mRNA abundance using qRT-PCR. Except for ALAD and FBN1, all genes were involved in lipid metabolism. Genes varied considerably in their expression with protein levels varying at least three fold in adipose tissue samples. Measures of mRNA and protein levels obtained from the same samples were compared in order to assess the translation output from mRNAs to their respective protein levels. Overall the correlation between mRNA and protein translation was moderate (r=0.245) (Table 6) for samples of animals at slaughter. The average correlation between mRNA and protein varied widely, with GPD1 (0.841) exhibiting the highest (p<0.001) correlation, while the correlation between transcription and translation for FASN (-0.037) was poor (p>0.05). 

**Table 6:** Correlation between presence of mRNAs and their respective proteins.

**Gene ID**	**Description**	**R**	**p-value**
ACOX2	acyl-CoA oxidase 2, branched chain	-0.371	0.052
ALAD	aminolevulinate dehydratase	0.764	<0.001
ATP5C1	ATP synthase, H+ transporting, mitochondrial F1 complex, gamma polypeptide 1	0.178	0.364
ELOVL6	ELOVL fatty acid elongase 6	0.395	0.038
FABP4	fatty acid binding protein 4, adipocyte	0.233	0.232
FASN	fatty acid synthase	-0.037	0.850
FBN1	fibrillin 1	-0.240	0.219
GPD1	glycerol-3-phosphate dehydrogenase 1 (soluble)	0.841	<0.001
THRSP	thyroid hormone responsive	0.444	0.018

Correlation is significant at p<0.05

### Post-transcriptional regulation of protein expression in bovine adipose tissues

**Figure 4:**
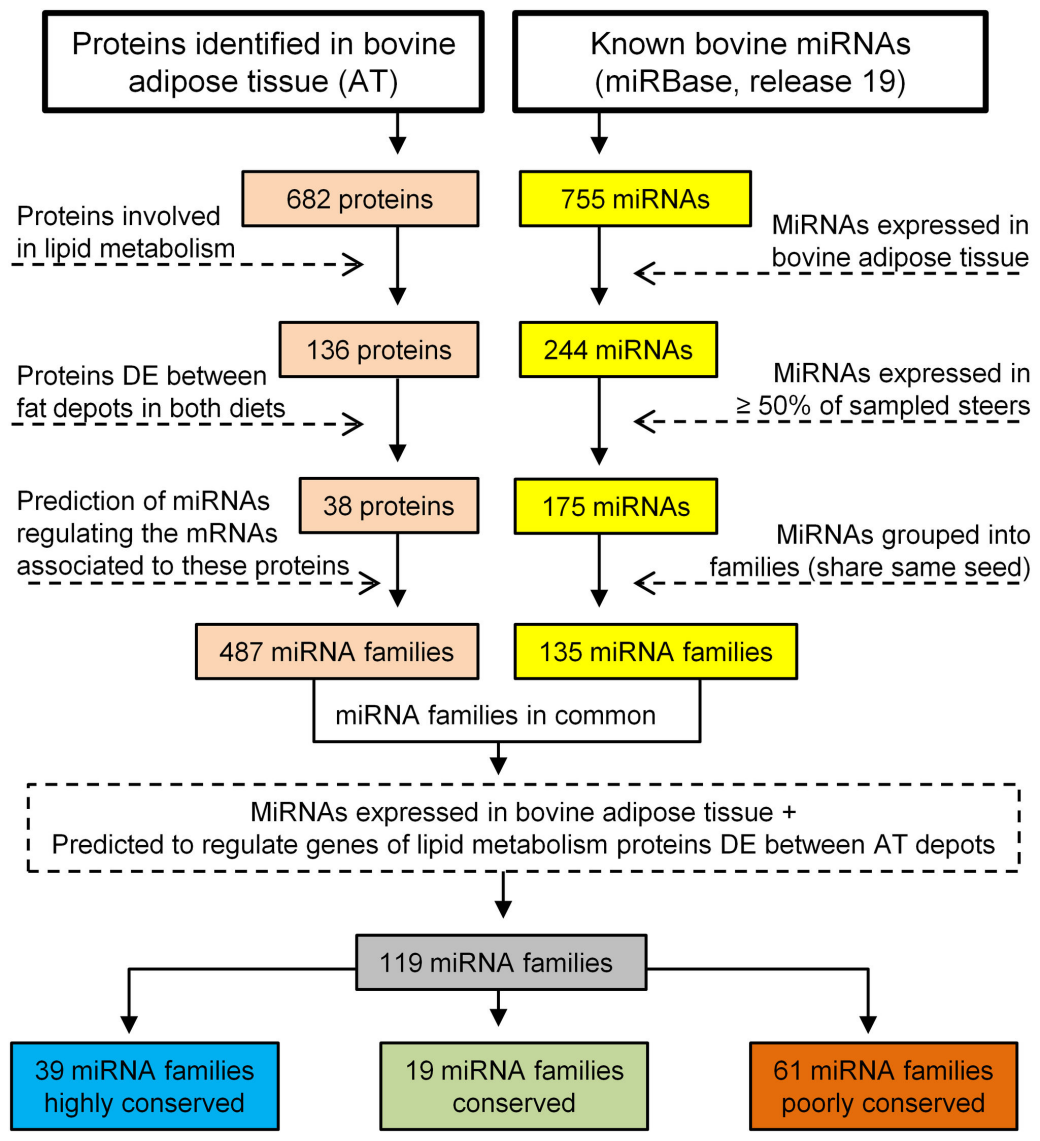
Integration of bovine miRNAs with proteins identified. The arrows with dotted lines represent the analysis performed to filter the miRNA and protein datasets. miRNA families highly conserved refer to miRNAs conserved across most vertebrates, miRNA families conserved are conserved across most mammals and miRNA families poorly conserved are not conserved beyond placental mammals.

miRNA-protein dataset integration was attempted in order to investigate how miRNAs might play a role in the regulation of lipid metabolism in bovine adipose tissue (Figure 4). TargetScan was used to predict miRNAs that bind to the 3’UTR of mRNAs associated to a subset of proteins identified in this study (n=38), corresponding to the proteins involved in lipid metabolism and differentially expressed between fat depots in steers fed both diets. A total of 487 miRNA families were predicted to regulate these genes. These miRNAs were compared to another set of miRNAs (135 miRNA families) obtained according to the following method. A total of 244 microRNAs from the same adipose tissues obtained from our previous microRNA microarray analysis [22] were filtered based on their frequency in the sampled steer population (cutoff ≥ 50% of animals) resulting in a total of 175 miRNAs. These miRNAs were grouped into families (miRNAs that share the same seed sequence), resulting in 135 miRNA families. (TargetScan www.targetscan.org/cgi-bin/targetscan/mirna_families.cgi?db=vert_61).

**Figure 5:**
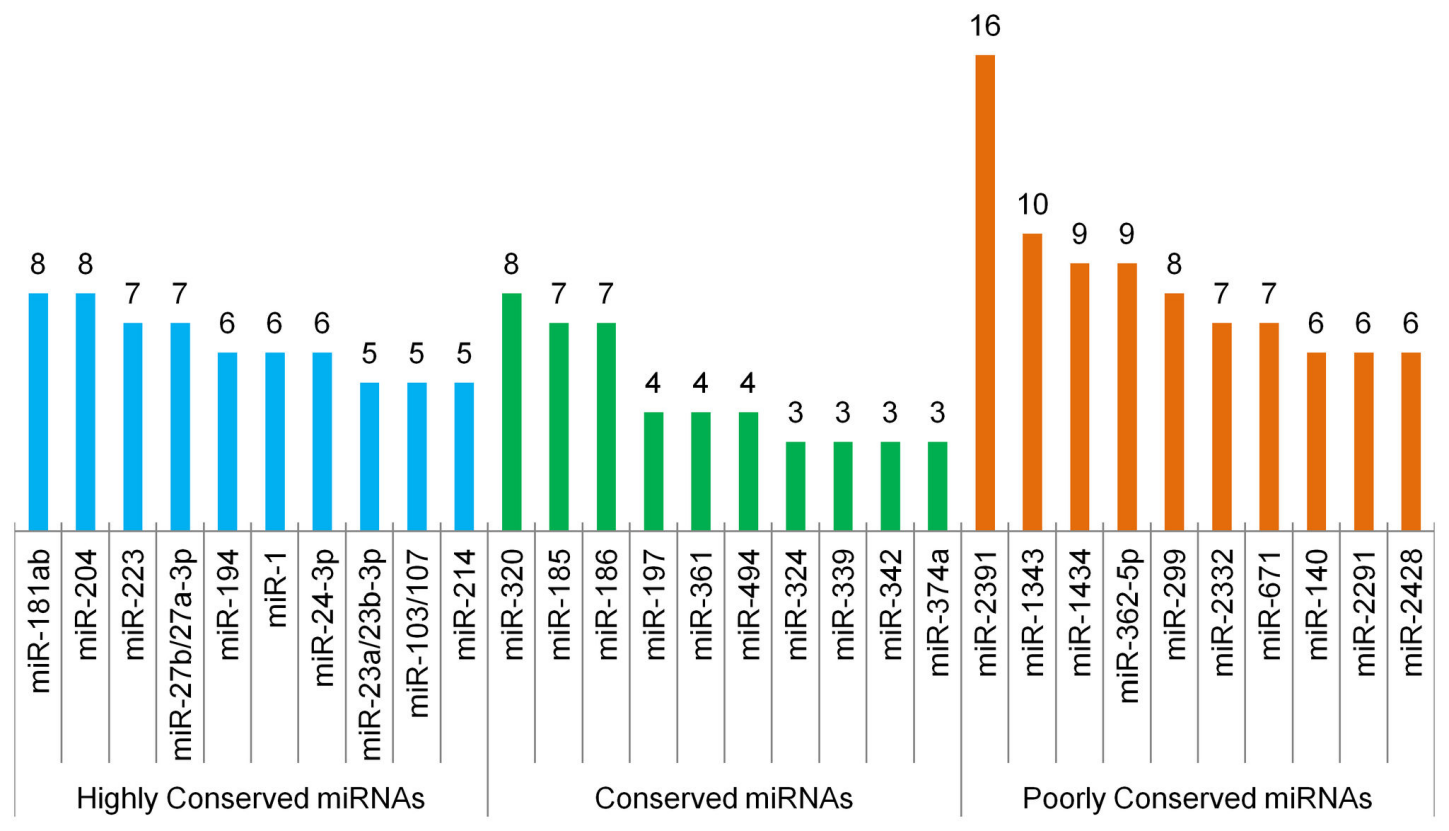
Top 10 miRNAs by conservation category predicted to regulate DE proteins between adipose depots involved in lipid metabolism. The columns indicate the number of proteins each miRNA is predicted to regulate. miRNA families highly conserved refer to miRNAs conserved across most vertebrates, miRNA families conserved are conserved across most mammals and miRNA families poorly conserved are not conserved beyond placental mammals.

 The comparison of the first dataset (487 miRNAs families) with the second (135 miRNA families) resulted in 119 miRNA families in common classified into different conservation status: highly conserved (conserved across most vertebrates), conserved (conserved across most mammals) and poorly conserved (conserved not beyond placental mammals). These miRNA families were found to have at least one representative expressed in bovine adipose tissue [22]. Furthermore, they were predicted to regulate the mRNA of 38 genes involved in lipid metabolism that were found to code for DE proteins associated with subcutaneous and visceral adipose depots. A total of 8 miRNAs out of 119 miRNAs families were predicted to target more than 20% of the 38 genes transcripts (Figure 5). Table S2 lists the 38 genes involved in lipid metabolism genes with the respective miRNAs predicted to target them and detected in bovine adipose tissue, and Table S3 lists shows the conservation status for the 119 bovine miRNA families and the number of predicted targets for each of them.

## Discussion

Adipose tissue is dynamic and known to play an important role in energy homeostasis. Different fat depots are known to display distinct metabolic characteristics, such as distinctive gene expression profiles, and thus likely have distinctive physiology [27,28]. Additionally, the function and regulation of adipose tissue may be affected by many other factors, such as diet [29], age [30] and stress [31]. Previous studies attempted to characterize the molecular regulation of bovine adipose tissue at the transcript level under different dietary regimens [32], adiposity conditions [33] and between breeds of cattle [12]. However, it is known that mRNA expression profiles may not provide the best representation of phenotype, considering that correlations between mRNA and protein expressions are often low [17-19]. Therefore to the best of our knowledge, this study is the first to examine the molecular biology of bovine adipose tissue (*in vivo*) using a high throughput proteomic based approach. 

In this study, 682 proteins differing in their cellular locations were characterized and quantified (Figure 1) demonstrating that our extraction procedure was capable of retrieving proteins from the main cellular compartments of adipose tissue. The diversity of protein types detected is an indication that the protein extraction was able to capture a balanced portion of the adipose tissue proteome. However, 682 proteins represent only a fraction of the total proteins expressed in bovine adipose tissue as 18,034 genes were previously reported to be expressed in the subcutaneous fat of beef cattle [12]. Future studies to improve the protein identification will provide vital information of protein expression in bovine adipose tissue. In addition, mature adipocytes are the main cell type in adipose tissue, however it is important to take into account that adipose tissue consists of different cells, including preadipocytes, macrophages, endothelial cells and stem cells. Therefore the results from this study do not refer exclusively to adipocytes, but to the biology of adipose tissue as a whole. 

Not surprisingly, the IPA core analysis predicted lipid metabolism as the major function of bovine adipose tissue, but proteins involved in many other functions (Figure 2) were also identified, supporting the role of adipose tissue in other metabolic activities such as an endocrine functions [1,2] as supported by the production of adiponectin. This adipokine, detected in our study (Table S1), is known as an important regulator of glucose and fatty acid metabolism in skeletal muscle [34]. Immune function proteins were represented as well (Table S1), for example with the presence of complement component 3 protein (C3), which is part of the immune system complement pathway [35]. 

All 682 proteins detected in this study were present in both subcutaneous and visceral adipose tissue from steers, regardless of diet. The presence of the same proteins in all groups suggests that they are involved in core functions in terms of the biological processes as predicted by their functional analysis (Figure 2). However, the activity status of these processes may be different among the groups as fat depot has a significant impact on the expression of most proteins and diet also affects protein expression to a lesser extent (Table 1 and Figure 3). More DE proteins were observed between adipose depots than between two diets suggesting that the molecular mechanism of adipogenesis differs between these depots. Downstream analysis supports this contention as demonstrated by the upregulated function of lipid synthesis and lipid oxidation in visceral fat tissue. Previous studies using bovine adipocyte culture [26] and human adipose tissue [36] together with our findings support that subcutaneous and visceral adipose tissue have differentially expressed proteins and are physiologically distinct [37]. Differentially expressed proteins between fat depots were involved with lipid metabolism, energy production and small molecule biochemistry of diet (Table 2). Therefore, it can be inferred that bovine subcutaneous and visceral fat differ in their lipid metabolism capabilities. Our results showing that lipid oxidation and synthesis of lipid functions are predicted to be significantly increased in visceral fat in control steers and lipid synthesis in steers fed the high fat diet (z-score > 2), concur that visceral adipose tissue is known to be more metabolically active than subcutaneous adipose tissue [36] and suggest that visceral fat is more involved in energy homeostasis while subcutaneous might be more involved with energy storage. However, dietary fat content also influenced the translation of a number of proteins despite having a lower impact on adipose tissue regulation. Its effect was more pronounced on subcutaneous adipose tissue as compared to visceral adipose suggesting that depots respond differently to the inclusion of fat in the diet. This finding may have implications to beef quality, as visceral fat is discarded, while subcutaneous fat is partially consumed with the muscles and seems to be more responsive to dietary fat. For instance, stearoyl-CoA desaturase (SCD) was more highly expressed in steers fed the high fat diet (flax supplemented). This enzyme converts saturated fatty acids into monounsaturated fatty acids such as stearic acid into oleic acid, which is the most abundant fatty acid in bovine adipose tissue [38]. SCD is important to bovine preadipocyte differentiation and its product (oleic acid) softens fat, a property that can impact beef quality [38]. A recent study on fatty acid profiles of the same bovine subcutaneous fat indeed revealed that flax seed supplementation increased alpha linoleic acid content [39], suggesting that altered SCD protein level may be an indicator of the beef quality and in the future this protein targeted nutritional management may improve the healthy fat component in beef product.

 Interestingly, steers fed the high fat diet had a downregulation of integrin alpha 6 (ITGA6), which was found to induce growth arrest in preadipocytes [40], which favours differentiation over proliferation. Therefore, both adipose tissues in steers fed the high fat diet may have been undergoing a more intense preadipocyte proliferation. Our previous study using the same steers showed that those fed the high fat diet expressed more miRNAs in adipose tissue than those fed the control diet [22], indicating that the higher fat content of the diet may increase the regulatory role of miRNAs in adipose tissue metabolism. There were less DE proteins between diets, yet these proteins are involved in important functions such as cellular development, cell morphology and lipid metabolism. A previous study [32] showed that dietary manipulation of fat content in bovine diets can impact the expression of several lipogenic genes, but our results did not find an extensive impact at the protein level.

Several proteins in this study had significant positive correlation with adiposity traits such as, subcutaneous fat thickness and adipocyte size (Tables 3 & 4). The fatty acid binding protein 5 (FABP5) and FABP4 were positively correlated with subcutaneous fat thickness and adipocyte size, respectively. FABP4 and FABP5 are associated with the terminal differentiation of adipocytes and are responsible for the intracellular transport of fatty acids [41,42]. These findings suggest that a higher expression of these proteins supports a higher degree of adiposity in this fat depot. NAD(P)H dehydrogenase quinone 1 (NQO1) was found to be the best marker for the differentiation process of swine adipocytes *in vitro*, being induced during adipogenesis [43] and in our study it was significantly positively correlated with subcutaneous fat thickness. This suggests that this protein is also associated with bovine adipogenesis although previous studies have revealed that molecular mechanisms of bovine adipocyte differentiation are different from that of porcine adipogenesis [44]. Transaldolase 1 protein (TALDO1) was also significantly correlated to adipocyte size. Interestingly, this protein was upregulated by 2 fold of magnitude in a study comparing subcutaneous fat of steers (castrated) and bulls (not castrated) [45]. The differential expression of this protein suggests that it might be involved in the heighted deposition of subcutaneous fat in steers as compared to bulls. Other proteins were significantly correlated with fat traits, but their function as related to adipogenesis is unknown.

Proteins are the final stage of gene expression in adipose tissue. Adipocyte protein levels may be regulated by several upstream mechanisms, including transcriptional regulatory factors [46], miRNAs [21], and epigenetics [47]. The upstream regulator analysis performed in this study predicted transcription factors that might possibly be involved in bovine adipose tissue regulation. Transcription factors play a fundamental role in the regulation of gene expression, impacting the protein output. Furthermore, they may account for a considerable portion of the genes and expressed proteins as illustrated by the finding in the human genome where they account for at least 6% of total protein coding genes [48]. It is important to point out that from the total proteins profiled in our study only two were transcription factors (~ 0.3% of total). It is known that transcription factors are expressed at much lower levels than non-transcription factor genes/proteins [48]. Therefore, transcription factors are much more difficult to quantify as compared to proteins expressed at higher levels. Therefore, this bioinformatics analysis provided important information about the expression of adipose transcription factors regulating DE proteins between subcutaneous and visceral adipose depots, which would not be obtained with LC-MS/MS alone. Except for myocyte enhancer factor 2C (MEF2C), SAM pointed domain containing ets transcription factor (SPDEF), and T-box 5(TBX5), the functions of all other identified TFs can involve aspects of lipid metabolism or adipocyte development (Table 5) including peroxisome proliferator activated receptor alpha (PPAR-α), and the adipogenesis master regulator peroxisome proliferator activated receptor gamma (PPAR-γ) [14]. Both PPAR-γ and PPAR-α can regulate more than 10% of the differentially expressed proteins between adipose depots. This shows their crucial roles to the specific regulation of each fat depot leading to the unique physiological characteristics of each adipose site. 

Transcription factors regulate the levels of mRNA as they control the pace of transcription, but they do not alter the translation of proteins from mRNAs. Several reports describe an overall modest correlation between mRNA and protein expression [17-19] and our results with a weak correlation of 0.245 are in agreement (Table 6). This suggests that mRNA levels may not be good predictors of protein expression for all genes and therefore not an accurate predictor of phenotype. In this context, miRNAs may be considered as one of the key post-transcriptional regulators, especially given the magnitude of miRNAs expressed in bovine adipose tissue [22,49,50]. miRNAs are likely to play an important fine-tuning role in bovine adipogenesis by binding to mRNA targets and regulating their availability to translation which modulates gene expression and may reduce undesired fluctuations in proteins levels [24]. Our computational prediction identified a large number of miRNAs that may contribute to the physiological differences between bovine subcutaneous and visceral fat by targeting the transcripts of DE proteins between fat depots. Among the top 10 highly conserved miRNAs (Figure 5), miR-103/107 [51,52] and miR-27b/27a-3p [53,54] families are well known for their roles in the regulation of adipogenesis, a function that may be universal in most vertebrates. Several poorly conserved miRNAs were predicted to regulate transcripts originating DE proteins. Three bovine specific miRNAs (miR-2391, miR-1434 and miR-2332) were among the top 10 poorly conserved miRNAs (Figure 5), suggesting the potential bovine specific post regulatory mechanisms in adipogenesis. miR-2391 was the miRNA with the highest regulatory potential over the set of genes associated with proteins involved in lipid metabolism that were differentially expressed between fat depots. It is worth considering that the status of bovine specific miRNAs in miRBase [55] may be temporary as these miRNAs might be shared among other ruminant species that have not been reported yet.

In conclusion, the results obtained from this study revealed that the profile of the bovine adipose proteome differs between fat depots, indicating important functional and physiological differences such as a higher metabolic activity of visceral fat. This finding might indicate a more pronounced role of subcutaneous fat in energy storage while visceral might be more active in lipid metabolism and energy balance. Subcutaneous fat was more responsive to dietary fat in terms of DE proteins and that has potential to be explored as a strategy to improve fat quality through diet manipulation. Bovine adipogenesis is a complex biological process in which several transcription factors are predicted to regulate gene expression at transcription level. Besides, miRNAs may also play an important role in post transcriptional regulation as protein output from translation was not consistent with mRNA levels. Several miRNAs expressed in bovine adipose tissue were predicted to regulate genes coding DE proteins associated to fat depots, suggesting their roles in bovine adipogenesis. These results improve our understanding on adipogenesis and may help the development of feeding strategies to manipulate adiposity in beef cattle, which is an important aspect not only to meat quality and animal production but also to human health. 

## Materials and Methods

### Animal study and sample collection

A total of 16 British-continental steers (12 month old) were used in this experiment. Steers were selected based on similar body weight (~ 456 kg) and housed in individual pens at the Lethbridge Research Centre. They received feed and water *ad libitum*. The steers were fed experimental diets for approximately 14 weeks. The control diet contained 2.7% fat (Control group, n=8) and the high fat diet contained 7.1% fat (High fat group, n=8). Fat content was increased by including 10% flaxseed in the diet as described in Table S4. Throughout the experiment several performance measures were recorded including body weight gain, feed intake, feed conversion ratio and carcass traits including cutability, backfat thickness and adipocyte size. Growth performance results are reported elsewhere by He et al. [39,56]. Adipose tissue samples were collected from animal carcasses after slaughter, immediately frozen in liquid nitrogen, and kept at -80°C until analyzed. Subcutaneous fat was collected from the backfat depot, close to the region of the last thoracic vertebrae and visceral fat was collected around the kidneys. The study was approved by the Animal Care Committee of Lethbridge Research Centre, Agriculture Agri-food Canada with ACC# 0930.

### Measurement of adipocytes size

Subcutaneous adipose tissue was collected by biopsy, with a portion of the sample placed in warm saline solution and transported to the laboratory and processed immediately after sampling. Tissues were cut into small pieces of approximately 80 mg and fixed with 1 mL of 5% osmium tetroxide [57]. After removal from the osmium tetroxide solution, fixed tissues were placed in 8 mol/L urea in physiologic saline (NaCl 0.9%) to soften the tissue in order to isolate adipocytes. The cells were then washed with saline and transferred to a 24 well plate for microphotography using an inverted microscope (Olympus CKX41, Olympus, Japan) with a digital camera (Moticam 2300, Motic China Group Co., Ltd., China). The diameter of cells was determined by computer image analysis using software of Motic Images Plus 2.0 ML as described by He et al [58]. 

### Protein extraction and total protein quantification

Tissue samples stored at -80°C were ground using liquid nitrogen. Protein extraction was performed for each sample by homogenizing 100 mg of ground adipose tissue with 1mL of 2-D protein extraction buffer-V with diluent II (Urea (< 8 M), Thiourea (< 5 M), and CHAPS (< 10%) (GE Healthcare, Uppsala, Sweden) added with DTT (40mM), using a Precellys^®^24 tissue homogenizer (Bertin Technologies, Saint-Quentin, France). The homogenate was centrifuged at 17,000 x g for 30 min at 4°C and the supernatant was transferred to new tubes avoiding the lipid layer formed. Total protein quantification was performed after extraction using RC DC (reducing agent compatible and detergent compatible) Protein Assay based on Lowry method (Bio-Rad, Hercules, CA, USA) according to manufacturer instructions in order to ensure adequate protein quantity for downstream applications

. 

### Label free LC-MS/MS quantification

Thirty µg of total protein from each sample was subjected to Label-free quantification LC-MS/MS [59] at the Mass Spectrometry (MS) and Proteomics Resource of the W.M. Keck Foundation Biotechnology Resource Laboratory, Yale University. Details on LC-MS/MS analysis are described in Protocol S1. Briefly, the proteins were firstly precipitated using methanol/chloroform and dissolved in 8M urea/0.4M ammonium bicarbonate (pH=8.0) and DTT (45mM), incubated at 37°C for 20 min and cooled to room temperature. Following incubation with 100mM iodoacetamide (IAN) for 20 min. The protein samples were digested with 2µg of Lys C incubated for 5 h, followed by 2µg of trypsin and incubated overnight at 37°C. LC-MS/MS was performed on a LTQ Orbitrap XL (Thermo Scientific, Waltham, MA, USA) equipped with a Waters nanoAcquity UPLC system and used a Waters Symmetry® C18 180µm x 20mm trap column and a 1.7 µm, 75 µm x 250 mm nanoAcquity™ UPLC™ column (35°C) for peptide separation. A total of 0.2ug of sample per run was used and samples were randomized with 2 blanks after each run. Each sample was run in duplicate. LTQ Orbitrap XL acquired MS using 1 microscan, and a maximum inject time of 900ms followed by three data dependant MS/MS acquisitions in the ion trap [60] with a total cycle time for both MS and MS/MS acquisition of 2.4 sec. Data analysis was done using Progenesis LC-MS software (Nonlinear Dynamics Ltd., New Castle, U.K) (www.nonlinear.com). First, the acquired spectra were imported to the software. 1 sample run was selected as a reference while the others were automatically aligned to that run to minimize retention time variability between runs. All runs were selected for detection with an automatic detection limit. A normalization factor was then calculated for each run to account for differences in sample loads among injections. The MSMS were exported for Mascot database searching and results imported into the Progenesis LCMS software, where search hits were assigned to corresponding features.

### Database search and protein identification

The data were processed with Progenesis LCMS which provided the .mgf files that were searched using Mascot search algorithm version 2.2.0. (Matrix Science Inc., London, U.K.) [61] to identify proteins. The data was searched using the Uniprot database (http://www.uniprot.org), bovine taxonomy. The following search parameters were used: type of search (MS/MS Ion Search), enzyme (trypsin), variable modifications (carbamidomethyl (Cys), oxidation (Met)), mass values (monoisotopic), protein mass (unrestricted), peptide mass tolerance (± 25 ppm), fragment mass tolerance (± 0.6 Da), charge (+7), maximum missed cleavages (3), decoy (yes), and instrument type (ESI-TRAP). 

### RNA extraction

Total RNA was extracted from frozen (-80°C) ground adipose tissue. Homogenization of the fat tissue samples was performed using a Precellys^®^24 tissue homogenizer with TRIZOL® (TRI reagent, Invitrogen, Carlsbad, CA, USA) and RNA was extracted following the manufacturer’s instructions for samples with high fat content. The concentration of total RNA was measured using the NANODROP® spectrophotometer ND-1000 (Thermo Scientific, Waltham, MA, US) and RNA integrity was measured using the Agilent 2100 BIOANALYZER® (Agilent Technologies Deutschland GmbH, Waldbronn, Germany). RNA with integrity number (RIN) > 7.8 was used for qRT-PCR analysis.

### mRNA expression validation by qRT-PCR

Candidate mRNAs were selected based on label free protein quantification data for qRT-PCR validation. First strand was obtained from total RNA for each sample using random primers and reverse transcription reagents (Invitrogen, Carlsbad, CA, USA) according to manufacturer’s guidelines. Each PCR reaction (20µL) consisted of 2ng of template cDNA, 2× SYBR Green I Master Mix buffer (10 μL, Applied Biosystems, Foster City, CA), and 300 nM forward and reverse primers.

Fluorescence signal was detected with an ABI STEPONEPLUS Real-time PCR System detector® (Applied Biosystems) using the following conditions: 2 min at 50°C, 10 min at 95°C, 40 cycles of 15 s at 95°C and 1 min at 60°C. A total of 28 samples, 7 samples from each fat depot – diet combination were used for qRT-PCR analysis, with a total of 3 technical replicates per reaction. Beta-actin was used as reference gene in this study due to its stable expression among all animals and treatments. Primers were manufactured by Invitrogen and sequences are shown in table S5. Gene expression was analyzed by relative quantification (delta delta Ct method).

### Bioinformatics analysis

Functional analysis for all proteins and DE proteins was performed using Ingenuity Pathway Analysis Package (IPA). Molecules from the dataset were mapped to the Ingenuity Knowledge Base (http://www.ingenuity.com) and associated with biological functions. Right-tailed Fisher’s exact test was used to calculate a p-value determining the probability that each biological function assigned to that data set was relevant. A p-value < 0.05 indicated that the function was significant to the dataset. The Downstream Effects Analysis (IPA^®^) was based on proteins differentially expressed between diets x depot combinations and determined if a biological function increased or decreased based on which proteins were involved along with their expression values. Z-scores >2 or <-2 indicated that the activity of a relevant function was significantly increased or decreased. Upstream regulator analysis aimed at predicting which upstream regulators (e.g. transcription factors) control the expression of a set of genes. This analysis was based on genes coding DE proteins between fat depots and transcription factors predicted with p<0.05 were relevant to the dataset. Activation status of TFs (increased or decreased expression) was based on the expression of the proteins involved and significance was represented as Z-score. Significance statements for data analyzed through IPA (Ingenuity® Systems, www.ingenuity.com) in functional analysis (p-value), downstream effect analysis (z-score), and upstream regulator analysis (p-value and Z-score) were calculated based on IPA algorithms.

### Statistical analysis

Effects of fat depot, diet and their interaction on protein expression were measured through Two-Way ANOVA with significance level at p<0.01. Proteins differentially expressed between treatments were selected based on least square means from Two-Way ANOVA (p<0.05) and a > 1.5 fold change. Correlations between individual measured fat traits (subcutaneous fat thickness and adipocyte size) and individual protein expression were calculated using Pearson correlation coefficient (R) with significance defined at p<0.05. The same was applied to correlations between mRNA and protein expression. Statistical analysis was performed with SAS software (v.9.0). 

## Supporting Information

Protocol S1 **Label Free Quantitation LCMS Methodology Report.**
(DOCX)

Table S1 **Protein annotation, normalized abundance by group and treatment effects.**
(XLSX)

Table S2 **miRNAs that target lipid metabolism genes whose protein were differentially expressed between adipose depots in both diets.**
(XLSX)

Table S3 **Number of predicted targets for the 119 bovine miRNA families and their conservation status.**
(XLSX)

Table S4 **Formulation and nutritional composition of Control and High fat diets.**
(XLSX)

Table S5 **Primer sequences.**
(DOCX)

## References

[1] Poulos SP, Hausman DB, Hausman GJ (2010) The development and endocrine functions of adipose tissue. Mol Cell Endocrinol 323: 20-34. doi:10.1016/j.mce.2009.12.011. PubMed: 20025936. 20025936

[2] Galic S, Oakhill JS, Steinberg GR (2010) Adipose tissue as an endocrine organ. Mol Cell Endocrinol 316: 129-139. doi:10.1016/j.mce.2009.08.018. PubMed: 19723556. 19723556

[3] Catenacci VA, Hill JO, Wyatt HR (2009) The Obesity Epidemic. Clin Chest Med 30: 415-444. doi:10.1016/j.ccm.2009.05.001. PubMed: 19700042. 19700042

[4] Nguyen T, Lau DCW (2012) The Obesity Epidemic and Its Impact on Hypertension. Can J Cardiol 28: 326-333. doi:10.1016/j.cjca.2012.07.549. PubMed: 22595448. 22595448

[5] McNeill SH, Harris KB, Field TG, Van Elswyk ME (2012) The evolution of lean beef: Identifying lean beef in today's U.S. Marketplace - Meat Science 90: 1-8. doi:10.1016/j.meatsci.2011.05.023. 21737208

[6] Wang Y, Beydoun MA (2009) Meat consumption is associated with obesity and central obesity among US adults. Int J Obes (Lond) 33: 621-628. doi:10.1038/ijo.2009.45. PubMed: 19308071. 19308071 PMC2697260

[7] FAO (2011) World Livestock 2011 – Livestock in food security. Rome, FAO.

[8] Dodson MV, Jiang Z, Chen J, Hausman GJ, Guan LL et al. (2010) Allied Industry Approaches to Alter Intramuscular Fat Content and Composition in Beef. Animals - Journal of Food Science 75: R1-R8. 20492190 10.1111/j.1750-3841.2009.01396.x

[9] Hausman GJ, Dodson MV, Ajuwon K, Azain M, Barnes KM et al. (2009) Board-INVITED REVIEW: The biology and regulation of preadipocytes and adipocytes in meat animals. J Anim Sci 87: 1218-1246. doi:10.2527/jas.2008-1427. PubMed: 18849378. 18849378

[10] Large V, Peroni O, Letexier D, Ray H, Beylot M (2004) Metabolism of lipids in human white adipocyte. Diabetes and Metabolism 30: 294-309. 15525872 10.1016/s1262-3636(07)70121-0

[11] Hosooka T, Noguchi T, Kotani K, Nakamura T, Sakaue H et al. (2008) Dok1 mediates high-fat diet-induced adipocyte hypertrophy and obesity through modulation of PPAR-[gamma] phosphorylation. Nat Med 14: 188-193. doi:10.1038/nm1706. PubMed: 18204460. 18204460

[12] Jin W, Olson EN, Moore SS, Basarab JA, Basu U et al. (2012) Transcriptome analysis of subcutaneous adipose tissues in beef cattle using 3' digital gene expression-tag profiling. J Anim Sci 90: 171-183. doi:10.2527/jas.2011-4229. PubMed: 21856901. 21856901

[13] Kirkland JL, Tchkonia T, Pirtskhalava T, Han J, Karagiannides I (2002) Adipogenesis and aging: does aging make fat go MAD? Exp Gerontol 37: 757-767. doi:10.1016/S0531-5565(02)00014-1. PubMed: 12175476. 12175476

[14] White UA, Stephens JM (2010) Transcriptional factors that promote formation of white adipose tissue. Mol Cell Endocrinol 318: 10-14. doi:10.1016/j.mce.2009.08.023. PubMed: 19733624. 19733624 PMC3079373

[15] Lefterova MI, Lazar MA (2009) New developments in adipogenesis. Trends Endocrinol Metab 20: 107-114. doi:10.1016/j.tem.2008.11.005. PubMed: 19269847. 19269847

[16] Basu U, Romao JM, Guan LL (2012) Adipogenic Transcriptome Profiling Using High Throughput Technologies. Journal of Genomics 1: 22-28. 10.7150/jgen.3781 PMC4091434 25031652

[17] Ghazalpour A, Bennett B, Petyuk VA, Orozco L, Hagopian R et al. (2011) Comparative Analysis of Proteome and Transcriptome Variation in Mouse. PLoS Genet 7: e1001393 PubMed: 21695224. 21695224 10.1371/journal.pgen.1001393 PMC3111477

[18] Gry M, Rimini R, Strömberg S, Asplund A, Pontén F et al. (2009) Correlations between RNA and protein expression profiles in 23 human cell lines. BMC Genomics 10: 365. doi:10.1186/1471-2164-10-365. PubMed: 19660143. 19660143 PMC2728742

[19] Schwanhäusser B, Busse D, Li N, Dittmar G, Schuchhardt J et al. (2011) Global quantification of mammalian gene expression control. Nature 473: 337-342. doi:10.1038/nature10098. PubMed: 21593866. 21593866

[20] Xie H, Sun L, Lodish HF (2009) Targeting microRNAs in obesity. Expert Opin Ther Targets 13: 1227-1238. doi:10.1517/14728220903190707. PubMed: 19650761. 19650761 PMC3197810

[21] Romao JM, Jin W, Dodson MV, Hausman GJ, Moore SS et al. (2011) MicroRNA regulation in mammalian adipogenesis. Exp Biol Med (Maywood) 236: 997-1004. doi:10.1258/ebm.2011.011101. PubMed: 21844119. 21844119

[22] Romao JM, Jin W, He M, McAllister T, Guan LL (2012) Altered MicroRNA Expression in Bovine Subcutaneous and Visceral Adipose Tissues from Cattle under Different Diet. PLOS ONE 7: e40605. doi:10.1371/journal.pone.0040605. PubMed: 22815773. 22815773 PMC3398999

[23] Ferland-McCollough D, Fernandez-Twinn DS, Cannell IG, David H, Warner M et al. (2012) Programming of adipose tissue miR-483-3p and GDF-3 expression by maternal diet in type 2 diabetes. Cell Death Differ, 19: 1003–12. PubMed: 22223106. 22223106 10.1038/cdd.2011.183 PMC3354052

[24] Ebert Margaret S, Sharp Phillip A (2012) Roles for MicroRNAs in Conferring Robustness to Biological Processes. Cell 149: 515-524. doi:10.1016/j.cell.2012.04.005. PubMed: 22541426. 22541426 PMC3351105

[25] Zhao YM, Basu U, Dodson MV, Basarb J, Guan L (2010) Proteome differences associated with fat accumulation in bovine subcutaneous adipose tissues. Proteome Sci 8: 14. doi:10.1186/1477-5956-8-14. PubMed: 20298566. 20298566 PMC2853513

[26] Rajesh RV, Heo G-N, Park M-R, Nam J-S, Kim N-K et al. (2010) Proteomic analysis of bovine omental, subcutaneous and intramuscular preadipocytes during in vitro adipogenic differentiation. Comp Biochem Physiol Part D Genomics Proteomics 5: 234-244. doi:10.1016/j.cbd.2010.06.004. PubMed: 20656571. 20656571

[27] Bjørndal B, Burri L, Staalesen V, Skorve J, Berge RK (2011) Different Adipose Depots: Their Role in the Development of Metabolic Syndrome and Mitochondrial Response to Hypolipidemic Agents. J Obes, 2011: 2011 PubMed: 21403826. 10.1155/2011/490650 PMC3042633 21403826

[28] Hishikawa D, Hong Y-H, Roh S-g, Miyahara H, Nishimura Y et al. (2005) Identification of genes expressed differentially in subcutaneous and visceral fat of cattle, pig, and mouse. Physiol Genomics 21: 343-350. doi:10.1152/physiolgenomics.00184.2004. PubMed: 15784696. 15784696

[29] Zhao S, Wang J, Song X, Zhang X, Ge C et al. (2010) Impact of dietary protein on lipid metabolism-related gene expression in porcine adipose tissue. Nutr Metab (Lond) 7: 6 PubMed: 20205889. 20205889 10.1186/1743-7075-7-6 PMC2827416

[30] Zhang L, Ebenezer PJ, Dasuri K, Fernandez-Kim SO, Francis J et al. (2011) Aging is associated with hypoxia and oxidative stress in adipose tissue: implications for adipose function. Am J Physiol Endocrinol Metab 301: E599-E607. doi:10.1152/ajpendo.00059.2011. PubMed: 21586698. 21586698 PMC3275102

[31] Hosogai N, Fukuhara A, Oshima K, Miyata Y, Tanaka S et al. (2007) Adipose Tissue Hypoxia in Obesity and Its Impact on Adipocytokine Dysregulation. Diabetes 56: 901-911. doi:10.2337/db06-0911. PubMed: 17395738. 17395738

[32] Joseph SJ, Pratt SL, Pavan E, Rekaya R, Duckett SK (2010) Omega-6 Fat Supplementation Alters Lipogenic Gene Expression in Bovine Subcutaneous Adipose Tissue. Gene Regul Syst Bio 4: 91-101. PubMed: 21072324. 10.4137/GRSB.S5831 PMC2976073 21072324

[33] Taniguchi M, Guan LL, Basarab JA, Dodson MV, Moore SS (2008) Comparative analysis on gene expression profiles in cattle subcutaneous fat tissues. Comp Biochem Physiol Part D Genomics Proteomics 3: 251-256. doi:10.1016/j.cbd.2008.06.002. PubMed: 20494844. 20494844

[34] Sweeney G (2011) Adiponectin action: a combination of endocrine and autocrine/paracrine effects. Frontiers in Endocrinology 2. 10.3389/fendo.2011.00062 PMC3355882 22649379

[35] Mayilyan KR (2012) Complement genetics, deficiencies, and disease associations. Protein and Cell 3: 487-496. PubMed: 22773339. 22773339 10.1007/s13238-012-2924-6 PMC4875391

[36] Pérez-Pérez R, Ortega-Delgado FJ, García-Santos E, López JA, Camafeita E, et al. (2009) Differential Proteomics of Omental and Subcutaneous Adipose Tissue Reflects Their Unalike Biochemical and Metabolic Properties. Journal of Proteome Research 8: 1682-1693. PubMed: 19714809. 19714809 10.1021/pr800942k

[37] Baglioni S, Cantini G, Poli G, Francalanci M, Squecco R et al. (2012) Functional Differences in Visceral and Subcutaneous Fat Pads Originate from Differences in the Adipose. Stem Cells - PLOS ONE 7: e36569. 22574183 10.1371/journal.pone.0036569 PMC3344924

[38] Smith SB, Lunt DK, Chung KY, Choi CB, Tume RK et al. (2006) Adiposity, fatty acid composition, and delta-9 desaturase activity during growth in beef cattle. Animal Science Journal 77: 478-486. doi:10.1111/j.1740-0929.2006.00375.x.

[39] He ML, Sultana H, Oba M, Kastelic JP, Dugan MER et al. (2012) Triticale Dried Distillers’ Grain Increases Alpha-Linolenic Acid in Subcutaneous Fat of Beef Cattle Fed Oilseeds. Lipids 47: 1209-1220. doi:10.1007/s11745-012-3720-z. PubMed: 23054550. 23054550

[40] Liu J, DeYoung SM, Zhang M, Zhang M, Cheng A et al. (2005) Changes in integrin expression during adipocyte differentiation. Cell Metab 2: 165-177. doi:10.1016/j.cmet.2005.08.006. PubMed: 16154099. 16154099

[41] Cristancho AG, Lazar MA (2011) Forming functional fat: a growing understanding of adipocyte differentiation. Nat Rev Mol Cell Biol 12: 722-734. doi:10.1038/nrm3198. PubMed: 21952300. 21952300 PMC7171550

[42] Samulin J, Berget I, Lien S, Sundvold H (2008) Differential gene expression of fatty acid binding proteins during porcine adipogenesis. Comp Biochem Physiol B Biochem Mol Biol 151: 147-152. doi:10.1016/j.cbpb.2008.06.010. PubMed: 18621139. 18621139

[43] Monaco E, Bionaz M, Rodriguez-Zas S, Hurley WL, Wheeler MB (2012) Transcriptomics Comparison between Porcine Adipose and Bone Marrow Mesenchymal Stem Cells during In Vitro Osteogenic and Adipogenic. Differentiation - PLOS ONE 7: e32481. doi:10.1371/journal.pone.0032481. 22412878 PMC3296722

[44] Taniguchi M, Guan LL, Zhang B, Dodson MV, Okine E et al. (2008) Adipogenesis of bovine perimuscular preadipocytes. Biochem Biophys Res Commun 366: 54-59. doi:10.1016/j.bbrc.2007.11.110. PubMed: 18060854. 18060854

[45] Zhang Q, Lee H-G, Han J-A, Kang SK, Lee NK et al. (2012) Differentially expressed proteins associated with myogenesis and adipogenesis in skeletal muscle and adipose tissue between bulls and steers. Mol Biol Rep 39: 953-960. doi:10.1007/s11033-011-0821-3. PubMed: 21594731. 21594731

[46] Rosen ED, MacDougald OA (2006) Adipocyte differentiation from the inside out. Nat Rev Mol Cell Biol 7: 885-896. doi:10.1038/nrm2066. PubMed: 17139329. 17139329

[47] Fujiki K, Kano F, Shiota K, Murata M (2009) Expression of the peroxisome proliferator activated receptor gamma gene is repressed by DNA methylation in visceral adipose tissue of mouse models of diabetes. BMC Biol 7: 38. doi:10.1186/1741-7007-7-38. PubMed: 19589179. 19589179 PMC2715379

[48] Vaquerizas JM, Kummerfeld SK, Teichmann SA, Luscombe NM (2009) A census of human transcription factors: function, expression and evolution. Nat Rev Genet 10: 252-263. doi:10.1038/nrg2538. PubMed: 19274049. 19274049

[49] Jin W, Dodson MV, Moore SS, Basarab JA, Guan LL (2010) Characterization of microRNA expression in bovine adipose tissues: a potential regulatory mechanism of subcutaneous adipose tissue development. BMC Mol Biol 11: 29. doi:10.1186/1471-2199-11-29. PubMed: 20423511. 20423511 PMC2874793

[50] Jin W, Grant JR, Stothard P, Moore SS, Guan LL (2009) Characterization of bovine miRNAs by sequencing and bioinformatics analysis. BMC Mol Biol 10: 90. doi:10.1186/1471-2199-10-90. PubMed: 19758457. 19758457 PMC2761914

[51] Trajkovski M, Hausser J, Soutschek J, Bhat B, Akin A et al. (2011) MicroRNAs 103 and 107 regulate insulin sensitivity. Nature 474: 649-653. doi:10.1038/nature10112. PubMed: 21654750. 21654750

[52] Xie H, Lim B, Lodish HF (2009) MicroRNAs Induced During Adipogenesis that Accelerate Fat Cell Development Are Downregulated in Obesity. Diabetes 58: 1050-1057. doi:10.2337/db08-1299. PubMed: 19188425. 19188425 PMC2671055

[53] Karbiener M, Fischer C, Nowitsch S, Opriessnig P, Papak C et al. (2009) microRNA miR-27b impairs human adipocyte differentiation and targets PPAR[gamma]. Biochem Biophys Res Commun 390: 247-251. doi:10.1016/j.bbrc.2009.09.098. PubMed: 19800867. 19800867

[54] Kim SY, Kim AY, Lee HW, Son YH, Lee GY et al. (2010) miR-27a is a negative regulator of adipocyte differentiation via suppressing PPAR[gamma]. Expression - Biochemical and Biophysical Research Communications 392: 323-328. doi:10.1016/j.bbrc.2010.01.012. 20060380

[55] miRBASE (2012) The miRBase Sequence Database -- Release 19. Release 19 ed: miRBASE. pp. miRBASE: the microRNA database

[56] He ML, Hernandez-Calva LM, McAllister TA, Aalhus JL, Dugan MER et al. (2012) Inclusion of triticale dried distiller grains and flaxseed in feedlot cattle diets increases alpha-linolenic acid in beef without affecting carcass or meat quality traits. Journal of Animal Science 90, Suppl. 3: 600-600.

[57] Cartwright AL (1987) Determination of adipose tissue cellularity In: Hausman GJ Martin RJ Biology of the adipocyte. New York, NY: Van Nostrand Reinhold pp. 229–254.

[58] He ML, Sharma R, Mir PS, Okine E, Dodson MV (2010) Feed withdrawal abate regimens lipodystrophy and metabolic syndrome symptoms, such as glucose tolerance, are associated with the diameter of retroperitoneal adipocytes in rats. Nutr Res 30: 125-133. doi:10.1016/j.nutres.2009.09.009. PubMed: 20226998. 20226998

[59] Old WM, Meyer-Arendt K, Aveline-Wolf L, Pierce KG, Mendoza A et al. (2005) Comparison of Label-free Methods for Quantifying Human Proteins by Shotgun. Proteomics - Molecular and Cellular Proteomics 4: 1487-1502. doi:10.1074/mcp.M500084-MCP200. 15979981

[60] Bordner K, Au E, Carlyle B, Duque A, Kitchen R et al. (2011) Functional genomic and proteomic analysis reveals disruption of myelin-related genes and translation in a mouse model of early life neglect. Frontiers in Psychiatry 2. 10.3389/fpsyt.2011.00018 PMC3098717 21629843

[61] Hirosawa M, Hoshida M, Ishikawa M, Toya T (1993) MASCOT: multiple alignment system for protein sequences based on three-way dynamic programming. Comput Appl Biosci 9: 161-167. PubMed: 8481818. 8481818 10.1093/bioinformatics/9.2.161

