# Supplementary material for: Elucidation of Molecular Mechanisms of Physiological Variations between Bovine Subcutaneous and Visceral Fat Depots under Different Nutritional Regimes
Source: PLoS One. 2013 Dec 9;8(12):e83211. doi: 10.1371/journal.pone.0083211 (PMC3857301; doi:10.1371/journal.pone.0083211)
Supplement: Protocol S1 — Label Free Quantitation LCMS Methodology Report. (DOCX) [file pone.0083211.s001.docx]

**Protocol S1. Label Free Quantitation LCMS Methodology Report**

**Label Free Quantitation LCMS Methodology Report**

**WM Keck Foundation Biotechnology Resource Laboratory**

**Yale University, New Haven, CT 06511, USA**

**Client**: Josue Romao and Leluo Guan

**Sample Source**: bovine fat tissue samples from Control Group (C) and High Fat Group (HF), within each group from subcutaneous adipose tissue (SAT) or visceral adipose tissue (VAT) origin

**Sample Preparation**

1. removed 30 ug of each and performed Methanol/Chloroform precipitation

2. Dissolve protein pellets in 20ul 8M urea/0.4M ammonium bicarbonate

3. Check pH = 8.0

4. Add 5ul 45mM DTT to each sample- vortex, spin

5. Incubate 37 degrees for 20 minutes.

6.Cool to room temperature

7. Add 5ul 100mM IAN

8. Incubate RT in the dark 20 minutes

9. Add 44ul water to each sample

10. add 2ug of Lys C and incubate for 5 hours

11. Add 2ug trypsin to each

12. Incubate 37 degrees overnight

13. Desalt digest with Micro-spin columns dry in speedvac

14. an estimate 0.2ug was loaded on column

15. samples were randomized with 2 blanks after each run and each sample was run in duplicate

**LC-MS/MS on the LTQ Orbitrap**: The LTQ Orbitrap is equipped with a Waters nanoAcquity UPLC system, and uses a Waters Symmetry® C18 180µm x 20mm trap column and a 1.7 µm, 75 µm x 250 mm nanoAcquity™ UPLC™ column (35ºC) for peptide separation. [1] Trapping was done at 15µl/min, 99% Buffer A (100% water, 0.1% formic acid) for 1 min. Peptide separation was performed with a linear gradient over 90 minutes at a flow rate of 300 nl/min.

| A: 0.1% Formic Acid in Water | | |  |  |  |
| --- | --- | --- | --- | --- | --- |
| B: 0.075% Formic Acid in Acetonitrile | | | |  |  |
|  |  |  |  |  |  |
|  | Time | Flow | %A | %B | Curve |
| 1 |  | .300ul/min | 95 | 5 | 6 |
| 2 | 1 | .300ul/min | 95 | 5 | 6 |
| 3 | 9 | .300ul/min | 60 | 40 | 6 |
| 4 | 91 | .300ul/min | 15 | 85 | 6 |
| 5 | 95 | .300ul/min | 15 | 85 | 6 |
| 6 | 96 | .300ul/min | 95 | 5 | 6 |
| Total Run Time: 120 min | | |  |  |  |

MS was acquired in the Orbitrap using 1 microscan, and a maximum inject time of 900 ms followed by three data dependant MS/MS acquisitions in the ion trap (with precursor ions threshold of >3000); the total cycle time for both MS and MS/MS acquisition was 2.4 seconds. Peaks targeted for MS/MS fragmentation by collision induced dissociation (CID) were first isolated with a 2 Da window followed by normalized collision energy of 35%. Dynamic exclusion was activated where former target ions were excluded for 30 seconds. .

The data were processed with Progenesis LCMS (Nonlinear Dynamics, LLC.) and protein identification was searched using Mascot search algorithm (Matrix Science). See details below.

**Data Analyses:** Feature extraction, chromatographic/spectral alignment, data filtering, and statistical analysis were performed using Nonlinear Dynamics Progenesis LC-MS software (www.nonlinear.com). First, the .raw data files were imported into the program. A sample run was chosen as a reference (usually at or near the middle of all runs in a set), and all other runs were automatically aligned to that run in order to minimize retention time (RT) variability between runs. No adjustments are necessary in the m/z dimension due to the high mass accuracy of the spectrometer (typically <3ppm). All runs were selected for detection with an automatic detection limit. On the order of 32,000 features were detected . Features within RT ranges of 0-25 minutes and 87-120 minutes were filtered out, as were features with charge ≥ +8. A normalization factor was then calculated for each run to account for differences in sample load between injections. The experimental design was setup to group multiple injections from each run. The algorithm then calculates and tabulates raw and normalized abundances, max fold change, and Anova values for each feature in the data set. The features were tagged in sets based on characteristics such as MSMS>1, p<0.01, and p<0.01. The remaining MSMS were exported to an .mgf (Mascot generic file) for database searching (see below). After the Mascot search, an .xml file of the results is created, then imported into the Progenesis LCMS software, where search hits are assigned to corresponding features.

**Database searching:** The .mgf files created by the Progenesis LCMS are searched in-house using the Mascot algorithm (Hirosawa et al, 1993, version 2.2.0) for un-interpreted MS/MS spectra. The data was searched the Swiss protein database, Bovine taxonomy. Search parameters are as follows:

Type of search: MS/MS Ion Search

Enzyme: Trypsin/Lys-C (note that Lys C cuts after Lys and trypsin after both Lys and Arg)

Variable modifications: Carbamidomethyl (Cys), Oxidation (Met)

Mass values: Monoisotopic

Protein mass: Unrestricted

Peptide mass tolerance: ± 25 ppm

Fragment mass tolerance: ± 0.6 Da

Charge: +7

Max missed cleavages: 3

Decoy: Yes

Instrument type: ESI-TRAP

Using the Mascot database search algorithm, the Keck Facility considers a protein identified when Mascot lists it as significant and more than 2 unique peptides match the same protein. The Mascot significance score (similar to the “Confident scores” column in the Progenesis LCMS protein features spreadsheet) match is based on a MOWSE score and relies on multiple matches to more than one peptide from the same protein.

The Mascot search results were exported to an .xml file using a significance cutoff of p<0.05.

The Max fold change indicates the protein change over all of the patients and includes a column that indicated the highest change and the lowest change. This value will only be positive.

**Results Dissemination**: The Progenesis LCMS analyses were exported into an Excel spreadsheet containing the protein list which contains all the proteins identifications along with their corresponding scores and quantitation values (normalized and raw), Anova p-value, and number of peptides matched to each protein. A second Excel table with peptide features is available if required.

**Reference**

**1. Bordner K, Au E, Carlyle B, Duque A, Kitchen R, et al. (2011) Functional genomic and proteomic analysis reveals disruption of myelin-related genes and translation in a mouse model of early life neglect. Frontiers in Psychiatry 2.**
