# Supplementary material for: Elucidation of Molecular Mechanisms of Physiological Variations between Bovine Subcutaneous and Visceral Fat Depots under Different Nutritional Regimes
Source: PLoS One. 2013 Dec 9;8(12):e83211. doi: 10.1371/journal.pone.0083211 (PMC3857301; doi:10.1371/journal.pone.0083211)
Supplement: Table S5 — Primer sequences. (DOCX) [file pone.0083211.s006.docx]

| **Table S5. Primer sequences** | | | |
| --- | --- | --- | --- |
| **Gene** | **Description** | **Direction** | **Sequence 5’ - 3’** |
| ACOX2 | acyl-CoA oxidase 2, branched chain | Forward | CGCTTCGCACAGGTCCTACCA |
|  |  | Reverse | CGGGATTGGCGGCGGATGAC |
| ALAD | aminolevulinate dehydratase | Forward | AGGAGTGGCCAGGTACGGTGT |
|  |  | Reverse | GGCCTCGATAGCCGGGGAGT |
| ATP5C1 | ATP synthase, H+ transporting, mitochondrial F1 complex, gamma polypeptide 1 | Forward | GACCGTGCAGCCGCAATGGA |
|  |  | Reverse | ATACAGAGCCAAGGACCCCACTCC |
| ACTB | Beta-actin | Forward | CTAGGCACCAGGGCGTAA |
|  |  | Reverse | CCACACGGAGCTCGTTGTA |
| ELOVL6 | ELOVL fatty acid elongase 6 | Forward | CTAAGCAAAGCACCCGAACT |
|  |  | Reverse | CCAGCAACCATGTCCTTGTA |
| FABP4 | fatty acid binding protein 4, adipocyte | Forward | GGTAGGAAAATCAACCACCA |
|  |  | Reverse | GCAAACGTCATCCATTTCAA |
| FASN | fatty acid synthase | Forward | GCATCGCTGGCTACTCCTAC |
|  |  | Reverse | GTGTAGGCCATCACGAAGGT |
| FBN1 | fibrillin 1 | Forward | CAGAGCCAACCGGGCCAAGAG |
|  |  | Reverse | GGAATGCCGGCAAATGGGGACAA |
| GPD1 | glycerol-3-phosphate dehydrogenase 1 (soluble) | Forward | TCTGCCACCTTCTTGGAGAG |
|  |  | Reverse | CAGCTTCTGTCCATTCAGCA |
| THRSP | thyroid hormone responsive | Forward | CTACCTTCCTCTGAGCACCAGTTC |
|  |  | Reverse | ACACACTGACCAGGTGACAGACA |
